# Fibroblast-Derived Exosomes Contribute to Chemoresistance through Priming Cancer Stem Cells in Colorectal Cancer

**DOI:** 10.1371/journal.pone.0125625

**Published:** 2015-05-04

**Authors:** Yibing Hu, Chang Yan, Lei Mu, Kaiyu Huang, Xiaolan Li, Deding Tao, Yaqun Wu, Jichao Qin

**Affiliations:** 1 Department of Surgery, Tongji Hospital, Tongji Medical College, Huazhong University of Science and Technology, Wuhan, China; 2 Molecular Medicine Center, Tongji Hospital, Tongji Medical College, Huazhong University of Science and Technology, Wuhan, China; Spanish National Cancer Centre (CNIO), SPAIN

## Abstract

Chemotherapy resistance observed in patients with colorectal cancer (CRC) may be related to the presence of cancer stem cells (CSCs), but the underlying mechanism(s) remain unclear. Carcinoma-associated fibroblasts (CAFs) are intimately involved in tumor recurrence, and targeting them increases chemo-sensitivity. We investigated whether fibroblasts might increase CSCs thus mediating chemotherapy resistance. CSCs were isolated from either patient-derived xenografts or CRC cell lines based on expression of CD133. First, CSCs were found to be inherently resistant to cell death induced by chemotherapy. In addition, fibroblast-derived conditioned medium (CM) promoted percentage, clonogenicity and tumor growth of CSCs (i.e., CD133+ and TOP-GFP+) upon treatment with 5-fluorouracil (5-Fu) or oxaliplatin (OXA). Further investigations exhibited that exosomes, isolated from CM, similarly took the above effects. Inhibition of exosome secretion decreased the percentage, clonogenicity and tumor growth of CSCs. Altogether, our findings suggest that, besides targeting CSCs, new therapeutic strategies blocking CAFs secretion even before chemotherapy shall be developed to gain better clinical benefits in advanced CRCs.

## Introduction

Colorectal cancer (CRC) is one of the leading causes of cancer-related deaths worldwide, and its mortality has been steadily increasing over the past decades [1]. Chemotherapies have not dramatically improved clinical outcomes of patients with recurrent or metastatic CRC. A better understanding of mechanisms underlying resistance in CRC is imperative for the development of more effective therapeutic approaches that can benefit CRC patients.

CRC is heterogeneous, manifesting variegated cellular morphologies and histopathological presentations. Experimental evidence for the existence of cancer stem cells (CSCs) in CRC was recently shown using primary human CRC tumor samples [2, 3]. CSCs are hypothesized to be inherently resistant to chemotherapy. Recurrent CRCs upon chemotherapy treatment are frequently enriched for the cells expressing CSC markers such as ABCB5 [2, 4]. Nevertheless, the underlying mechanisms are still being defined.

Carcinoma-associated fibroblasts (CAFs) are intimately involved in tumor maintenance and progression. CAFs are also reported to play significant roles in regulating tumor sensitivity to a variety of chemotherapies, and targeting them dramatically decreases the chemoresistance [5, 6]. Here, we first confirm that chemotherapy preferentially targets non-CSCs due to cell autonomous resistance of CSCs, and further uncover that CAFs prime CSCs and increase drug resistance upon chemotherapy through CAF-derived exosomes.

## Material and Methods

### Ethics Statement

Colorectal adenocarcinoma tissue samples were obtained from patients that underwent surgical procedures within the Tongji Hospital of Tongji Medical College, Huazhong University of Science and Technology. Written informed consent was obtained from all research subjects and all protocols were approved by the Ethical Committee of Tongji Hospital, Tongji Medical College, Huazhong University of Science and Technology (IRB ID: 20141106) and were conducted according to the principles of the Declaration of Helsinki.

### Antibody and reagents

Monoclonal mouse anti-human CD133 and mouse anti-human EpCAM antibodies were purchased from Miltenyi Biotec (Headquarters, Germany). Anti-α-SMA antibody was obtained from Dako (Denmark). Anti-FAP was purchased from Abcam (Cambridge, UK) and anti-vimentin was purchased from Cell Signaling Technology (Danvers, MA). Anti-Wnt3a and anti-beta Actin were purchased from Santa Cruz Biotechnology (CA, USA). Alexa Fluor 488-conjugated goat anti-mouse lgG was obtained from Jackson ImmunoResearch (Pennsylvania, USA). GW4869, 5-fluorouracil (5-Fu) and oxaliplatin (OXA) were purchased from Sigma (St. Louis, USA). Matrigel was obtained from B.D. (Franklin Lakes, NJ).

### Cell lines and cell culture

Human colon cancer cells (SW620) and human fibroblast cells derived from normal colon tissues (18Co) were purchased from American Type Culture Collection (Manassas VA). Cancer-associated fibroblasts (CAFs) were isolated from colorectal cancer specimens. SW620 cells, 18Co cells and CAFs derived from primary tumors were cultured in DMEM media (Invitrogen, California, USA) supplemented with 10% FBS (Life technologies, NY, USA) in a 37°C humidified incubator with an atmosphere of 5% CO_2_ and 95% air.

### Preparation of single cell suspensions from tumors

Primary colorectal tumors or xenograft tumors were minced completely to the size of 1mm^3^ and then suspended in DMEM/F12 media (Invitrogen, California, USA) containing 1.5mg/ml collagenase Ⅳ (Invitrogen, California, USA), 20ug/ml hyaluronidase (Sigma, St. Louis, USA), 1% penicillin/streptomycin (Life technologies, NY, USA) and 1.25mg/ml amphotericin B (Sigma, St. Louis, USA) at 37°C for 1 hour. After digestion, tissues were washed with PBS and filtered through a 40μm mesh (BD Falcon, CA, USA). To eliminate red blood cells, the cells were incubated in red blood cell lysis buffer (eBioscience, California, USA) on ice for 10 minutes and washed twice with PBS. The cells were then resuspended in PBS for experiments.

### Isolation of CAFs and establishment of CRC xenograft tumors (XhCRC)

To isolate CAFs, single cells obtained from a female patient with Duke B colorectal adenocarcinoma were cultured in DMEM with 10% FBS. After incubated for 3 hours, the non-adherent cells were washed away with PBS, leaving adherent cells that mainly consisted of macrophages, epithelial cells, and fibroblasts. After cultured for several days, the macrophages and epithelial cells died off, leaving cells that were fibroblast-like and consistently α-SMA, vimentin and FAP positive. Primary fibroblast cultures were used for experiments up to passage 10. To establish CRC xenograft tumor model, single primary colorectal cancer cells derived from a female patient with Duke C colorectal adenocarcinoma were implanted into bilateral backs of female NOD/SCID mice.

### Fluorescence-activated cell sorting (FACS) and purification of CD133^+/-^ CRC cells

The FACS was performed according to the manufacturer’s instructions using a FACS Aria II Cell Sorter (BD, Biosciences, CA, USA). To separate CD133^+^ and CD133^-/lo^ cells in SW620 cells, cells were labeled with PE/APC-conjugated mouse anti-human CD133 antibodies. Generally, only the top (CD133^+^) and bottom (CD133^-/lo^) 10–20% cells were purified out. To separate CD133^+^ and CD133^-/lo^ cells in xenografts, XhCRC tumors were dissociated to single cells as described above and then stained with APC-conjugated mouse anti-human CD133 and PE-conjugated mouse anti-human EpCAM antibodies, and EpCAM^+^CD133^+^ and EpCAM^+^CD133^-/lo^ CRC cells were purified out.

### Cell death analysis

Cell death of CSCs and non-CSCs was assessed using the Cell Counting Kit-8 (Dojindo, Japan). Briefly, cells were seeded in complete medium at 3,000 cells/well in 96-well plates. After 12 hours post seeding, the cells were treated with either 5-Fu (1μM) or OXA (1μM). And 72 hours later, 10μl CCK-8 solution was added to each well. Then plates were incubated at 37°C for 1 hour, cell viability was determined by scanning with mircoplate reader at 450nm.

### Conditioned medium preparation

Fibroblasts were plated and cultured in DMEM/F12 media with 10% FBS for 24 hours, and then washed for three times with PBS and finally cultured in 3ml serum free DMEM/F12 media for 2 hours. Conditioned medium was collected and filtered through a 0.22-μm filter (Merck Millipore, Massachusetts, USA) to remove cellular debris.

### Isolation and characterization of exosomes released by fibroblasts

Exosomes were isolated from cultured fibroblasts by using Total Exosome Isolation Kit (Invitrogen, California, USA). In brief, 0.5ml of total exosome isolation reagent was added to each 1ml of filtered conditioned media and mixed well by inverting. After incubated overnight at 4°C, the mixture was centrifuged at 12,000×g for 70min at 4°C and all supernatant was removed by aspiration [7]. Exosome pellets were resuspended with a convenient volume of DMEM/F12 medium. Another exosome isolation method is performed by serial centrifugation as described previously [8]. Cellular supernatant was firstly centrifugated at 2,000 × g for 30 min, 10,000 × g for 40 min to deplete dead cells and cell debris. And then, exosomes and exosome-depleted soluble fractions were separated by ultracentrifugation at 100,000×g 4°C for 70 min.

The morphology and particle size of exosomes were characterized via a transmission electron microscopy (FEI Tecnai 20, Philips) operating at 160kV. Exosome proteins were extracted from exosomes using SDS lysis buffer (250nM Tris-HCL, pH 7.4, 2.5% SDS). Proteins were loaded on 10% SDS-polyacrylamide gels, electrophoretically transferred to nitrocellulose membranes. Mouse anti-human CD81 antibodies (Santa Cruz Biotechnology, Santa Cruz, CA, USA, clone: 1.3.3.22, 1:100) were used for immunoblotting. Protein bands were visualized using Super Signal West Femto Maximum Sensitivity Substrate (Thermo Scientific, Waltham, MA).

### Inhibition of exosome release

To further validate the effects of exosomes, exosome releasing was blocked by culturing with 10μM GW4869, a specific inhibitor for neutral sphingomyelinase 2 (nSMase2) [9]. After incubation for 24 hours, the medium that contained GW4869 was discarded and cells were washed by PBS for 3 times. Then fresh DMEM/F12 medium was added, and conditioned medium was harvested as described above.

### Sphere-formation assay

Basic procedures for sphere-formation assay were previously described [10]. Cells were resuspended in standard sphere-forming medium [DMEM/F12 (Invitrogen, California, USA) supplemented with 1× B27 serum substitute (Invitrogen, California, USA), 20ng/ml human recombinant epidermal growth factor and 20ng/ml basic fibroblast growth factor (Sigma,St. Louis, USA). CRC cells were plated at 300~500 cells/well in 24-well ultra-low attachment plates (Corning, Massachusetts, USA) with or without administration of 5-Fu (1μM) or OXA (1μM). For serial sphere-formation assays, the first generation spheres were harvested, disaggregated with 0.025% trypsin/EDTA, filtered through 40-μm mesh and re-plated as above. This process was repeated for up to 3 generations. When treated with chemotherapy, or/and CM/exosomes, chemotherapeutic agents or/and CM (200μl)/exosomes (equal to 200μl CM) were added every 2 days. After 5~14days, spheres with diameters ≥ 50μm were scored and shown as clonogenicity (%) in the figures.

### Animal study

Animal protocols were approved by University Committee for the Use and Care of Animals (UCUCA) at Tongji Medical College, Huazhong University of Science and Technology. In all experiments, viability of cells was confirmed by trypan blue exclusion test and cells were resuspended in 100μl PBS/Matrigel mixture (1:1 volume), followed by injection into the subcutaneous tissue of the left and right back area using a 27-gauge needle. SW620 cells were subcutaneously implanted into 4-week-old female balb/c-nu mice, and XhCRC cells were implanted into 4-week-old female NOD/SCID mice. Four to five mice were inoculated per group. CM (200μ) or exosomes (equal to 200μl CM) were then injected subcutaneously every 2 days. Simultaneously, all the mice were intraperitoneal injected with a chemotherapeutic agent, 5-Fu (100mg/kg body weight) or OXA (10mg/kg body weight) once a week. Tumors were monitored, and tumor volumes were examined every three days. After sacrificed, tumors were removed from mice and weighed to evaluate the tumor development. For limiting dilution assays, 100,000, 10,000, 1,000, and 100 purified CD133^+^ and CD133^-/lo^ CRC cells were implanted inimmunodeficient mice. Tumor-initiating frequency and statistical significance were evaluated with the Extreme Limiting Dilution Analysis (ELDA) ‘limdil’ function (http://bioinf.wehi.edu.au/software/elda/index.html).

### Immunofluorescence microscopy

Immunofluorescence microscopy was previously described [11].CAFs or XhCRC cells were cultured on 0.17-mm coverslips or glass-bottomed Petri dishes in monolayer overnight. Cells were fixed by 4% paraformaldehyde (PFA) for 10min at room temperature and followed by blocking in 5% (w/v) bovine serum albumin (BSA) in PBS buffer for 1 hour. Primary antibodies were diluted in 1:50 in 5% BSA. After incubated overnight at 4°C, cells were then rinsed three times with PBS and followed by incubation with Alexa Fluor 488-conjugated secondary antibodies (diluted 1:50 in 5% BSA) for 2 hours at room temperature. Finally, DAPI (4’,6-Diamidino-2-phenylindole, Sigma) was used to visualize cell nuclei for 10min at room temperature. Slides were examined under a confocal microscope (FV1000, Olympus).

### Dil-labeled exosomes transfer

Purified exosomes derived from 18Co cells were labeled with lipophilic fluoescentcarbocyanine dyes DiI according to the manufacturer’s protocol (Santa Cruz Biotecnology, CA, USA).18Co-exosomes were suspended in 100ul PBS and incubated with 1μl Dil for 15 min at 37°C, washed twice to remove excess dye, and incubated with SW620 cells at 37°C overnight.

### Lentiviral reporter assays

TCF/LEF reporter driving expression of GFP (TOP-GFP) lentivirus was purchased from SBO Medical Biotechnology Company, Shanghai, China. SW620 cells were infected with TOP-GFP lentivirus at MOI = 25 for 72 hours. For sphere formation assays, TOP-GFP^+^ and TOP-GFP^-^ fractions were sorted out by FACS and plated in ultra-low attachment plates as described above. To evaluated the effects of CM/exosomes on Wnt activity of CD133^+^ fractions, 50,000 CD133^+^ SW620 cells were infected with TOP-GFP lentivirus and plated in 6-well plates, followed by CM/exosomes treatment or plus chemotherapy. After 3 days incubation, expression of TOP-GFP was analyzed by flow cytometry.

### Statistical analysis

All data were presented as mean ± SD. In general, unpaired two-tailed Student’s *t*-test or two-way ANOVA test was performed on IBM SPSS Statistics 18 to compare differences between means of different treatment groups. A *P*-value <0.05 was considered to be statistically significant.

## Results

### CD133 identifies CSCs in a CRC cell line and xenograft derived from primary colorectal cancer tumor

**Fig 1:**
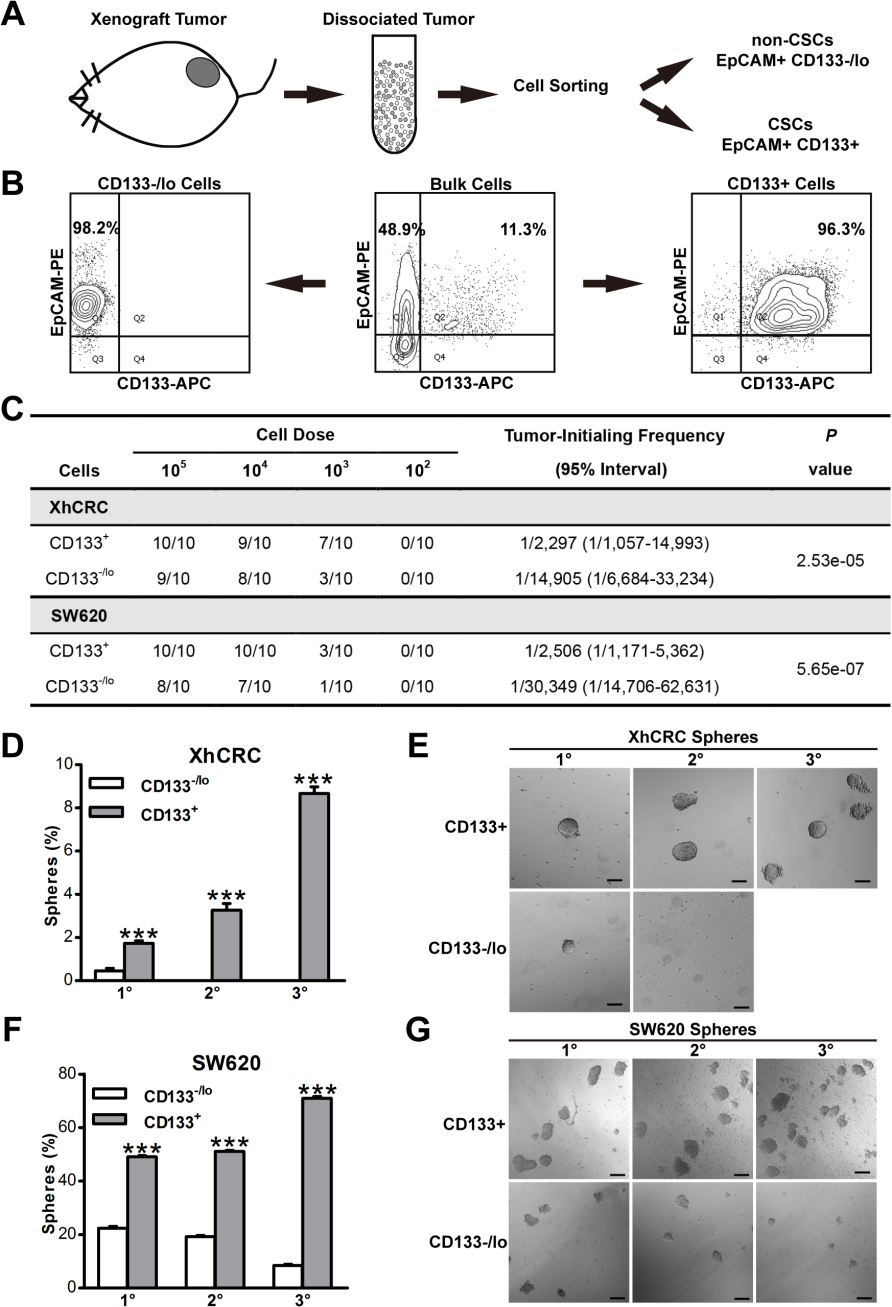
CD133 identifies CSCs in colorectal cancer. (A)Schematic of CD133^+^ and CD133^-/lo^ tumor cells sorting from dissociated colorectal xenograft tumor by FACS. (B) A representative example of post-sorting analysis of the sorted CD133^+^ and CD133^-/lo^ XhCRC cells.(C) Tumor-initiating frequency of CD133^+^ and CD133^-/lo^ CRC cells in immunodeficient mice (D-G)Serial sphere formation assays for purified CD133^+^ and CD133^-/lo^CRC cells (i.e., XhCRC and SW620). Spheres were enumerated (D, F) and representative images are shown (E, G).Scale bars, 100μm.***P< 0.001.

CSCs are a rare population within colorectal cancer which exhibit self-renewing and tumorigenic capacity [12]. The use of CD133, a surface maker, to enrich CSCs is essential to separate tumorigenic and non-tumorigenic cells [2]. We first established xenograft tumors (XhCRC) derived from a female patient with Duke C colorectal adenocarcinoma in female NOD/SCID mice. When xenograft tumors grew up, the tumors were processed into single cell suspensions, and we then sorted out two cell populations (i.e., EpCAM^+^CD133^+^ and EpCAM^+^CD133^-/lo^) based on expression of the epithelial marker (i.e., EpCAM) and the putative CSC marker (i.e., CD133) (Fig 1A).The purity of EpCAM^+^CD133^+^ and EpCAM^+^CD133^-/lo^ cells was both >96% (Fig 1B). To confirm that EpCAM^+^CD133^+^ cells enrich CSCs, we conducted limiting dilution assays. As expected, the EpCAM^+^CD133^+^ cells demonstrated higher tumor-generating capacity (*P*<0.001) (Fig 1C). Similarly, purified CD133^+^ SW620 cells, a widely-used CRC cell line, also initiated more (*P*<0.001) (Fig 1C). Consistent with LDAs, serial sphere-formation assays demonstrated that purified EpCAM^+^CD133^+^ cells were able to be passaged for at least three generations and showed an increased sphere-propagating capacity, whereas EpCAM^+^CD133^-/lo^ cells formed much less spheres in 1^o^ generation (*P*<0.001) and EpCAM^+^CD133^-/lo^ cell-initiated spheres aborted by 2^o^ generation (Fig 1D and 1E). In addition, purified CD133^+^ SW620 cells, were also exhibited a progressively increased sphere-propagating capacity when compared with CD133^-/lo^ SW620cells (*P*<0.001) (Fig 1F and 1G). These data indicated that CD133^+^ CRC cells possessed long-term clonogenicity in both xenograft tumors and SW620 cells, suggesting that CD133^+^ CRC cells, in our experimental system, may enrich for putative CSCs. For the sake of simplicity, from here onwards, we refer to EpCAM^+^CD133^+^ or CD133^+^ and EpCAM^+^CD133^-/lo^ or CD133^-/lo^ cells as CSCs and non-CSCs, respectively.

### CSCs exhibit cell-autonomous chemoresistance

**Fig 2:**
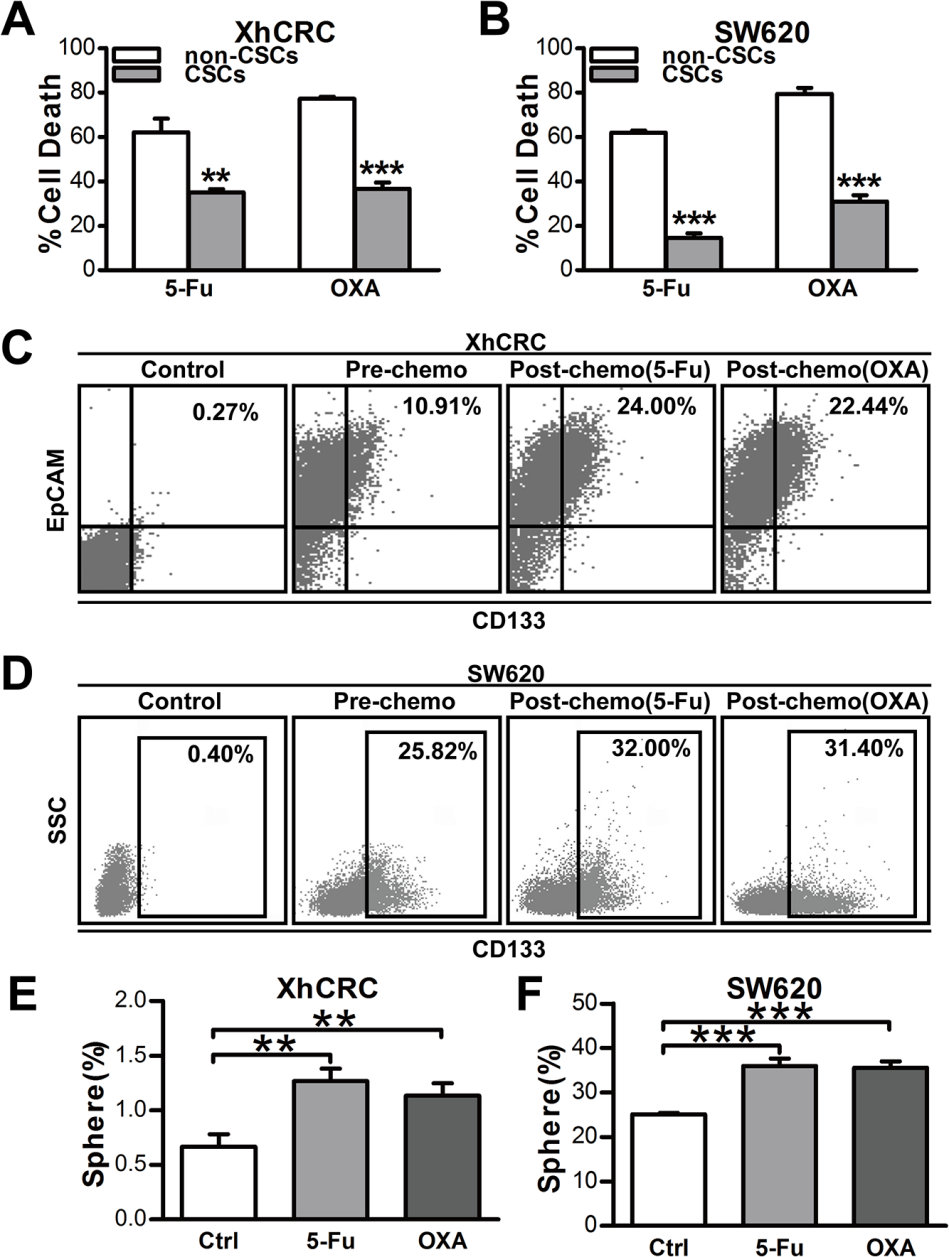
CSCs display cell-autonomous resistance to chemotherapy. (A, B) Cell death analysis of CSCs and non-CSCs from XhCRC or SW620 cells was assessed by CCK-8 activity assay upon chemotherapeutic treatment (5-Fu or OXA).**P< 0.01, ***P< 0.001. (C, D) Enrichment of CSCs in bulk cells from XhCRC (C) and SW620 cells (D) was assessed by FACS analysis based on CD133 expression upon chemotherapy. (E, F) Sphere-forming capacity of bulk cells (XhCRC or SW620 cells) pre-treated by chemotherapeutic agents or DMSO (Ctrl). **P< 0.01, ***P< 0.001.

As CSCs have been reported to be relatively resistant to chemotherapy [13–16], we interrogated the response of CSCs and non-CSCs to conventional chemotherapeutic agents (5-Fu or OXA) by CCK-8 activity assays. Indeed, in both XhCRC and SW620 cells, chemotherapy-induced cell death was significantly reduced in CSCs relative to non-CSCs (Fig 2A, *P*<0.01 in 5-Fu group, *P*<0.001 in OXA group, 2B, *P*<0.001), indicating that CSCs may be inherently resistant to chemotherapeutic agents. To determine the final cellular phenotype responsible for this difference, we treated bulk XhCRC and SW620 cells with 5-Fu or OXA for 3 days, and then detected the percentage of cells expressing CD133 by flow cytometry. As expected, CD133^+^ cells increased 0.5-1-fold after chemotherapeutic treatment (Fig 1D and 1E), and furthermore, the residual CRC cells (i.e., containing more CD133^+^ cells) also showed an increased sphere-forming capacity (Fig 2E, *P*<0.01, 2F, *P*<0.001), suggesting that chemotherapy, indeed, enriches CSCs in colorectal cancer through CSC-cell-autonomous chemoresistance.

### Fibroblast-derived conditioned medium primed SW620 CSCs and thus contributed to drug resistance

Recent studies have shown that tumor microenvironment mediates drug resistance [17],and carcinoma-associated fibroblasts (CAFs), as an important component of microenvironment, are deeply involved in chemotherapeutic resistance [18, 19]. Indeed, upon chemotherapy treatment, CAFs could be activated and maintained CSCs pool thus contributing to drug resistance [20]. However, a recent study has shown that CAFs, even without activation of chemotherapeutic agents, promote tumor stemness in colorectal cancer [21], implying that CAFs may prime CRC cells to increase tumor stemness before chemotherapy, and which may thus contribute to therapeutic resistance.

**Fig 3:**
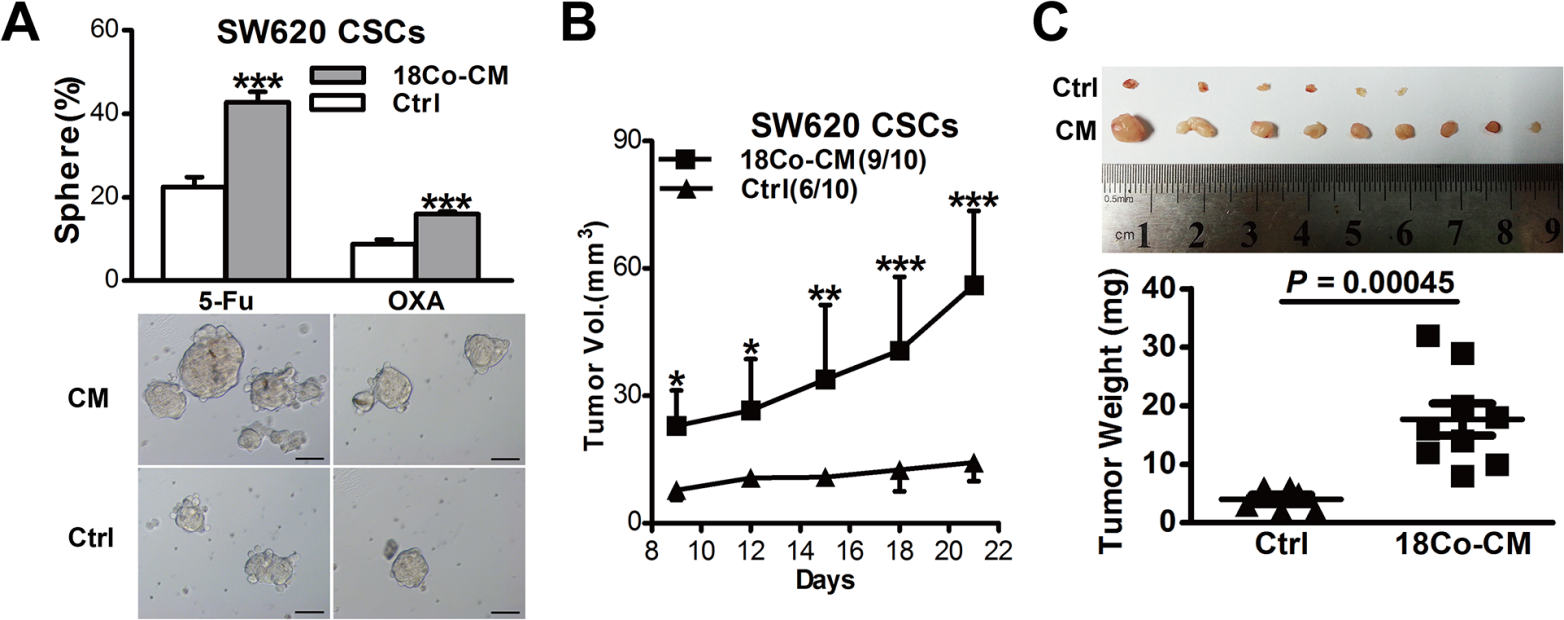
18Co cells prime SW620 CSCs to be more drug resistance via paracrine pathway. (A) Sphere-forming capacity of SW620 CSCs treated with 18Co-derived CM during chemotherapy (5-Fu or OXA). Representative microscopic images are shown. Scale bars, 100μm. (B, C) 18Co-derived conditioned medium affected tumor growth of SW620 CSCs in female Balb/c-nu mice treated with OXA. Tumor growth curves are shown in C, Shown in D are tumor weights and images at the end of experiments. Data are presented as mean ± SD; *P< 0.1; **P< 0.05; ***P< 0.001.

To determine whether fibroblasts prime CSCs thus contributing to drug resistance, we harvested conditioned medium from cultured 18Co (18Co-CM) without administration of chemotherapeutic agents. We then performed sphere formation assays with 18Co-CM in SW620 CSCs upon administration of 5-Fu or OXA. Expectedly, 18Co-CM treated SW620 CSCs generated more and larger spheres than SW620 CSCs in control medium (*P*<0.001) (Fig 3A). To further confirm the effects of 18Co-CM in vivo, we implanted 50,000 CSCs subcutaneously into bilateral backs of female balb/c-nu mice (n = 5), which were intraperitoneally injected with chemotherapeutic agents every week and also received injection of 18Co-CM or control medium every 2 days. In concordance with *in vitro* results, 18Co-CM treated mice manifested higher tumor incidence, faster tumor growth and larger tumors, illustrating that 18Co-CM is able to protect xenograft tumors from chemotherapy (Fig 3B and 3C).These findings suggest that fibroblast-derived conditioned medium without chemotherapy treatment may prime CSCs and thus promote chemoresistance in CRC.

### CAF-derived conditioned medium primed XhCRC CSCs and promoted chemoresistance

**Fig 4:**
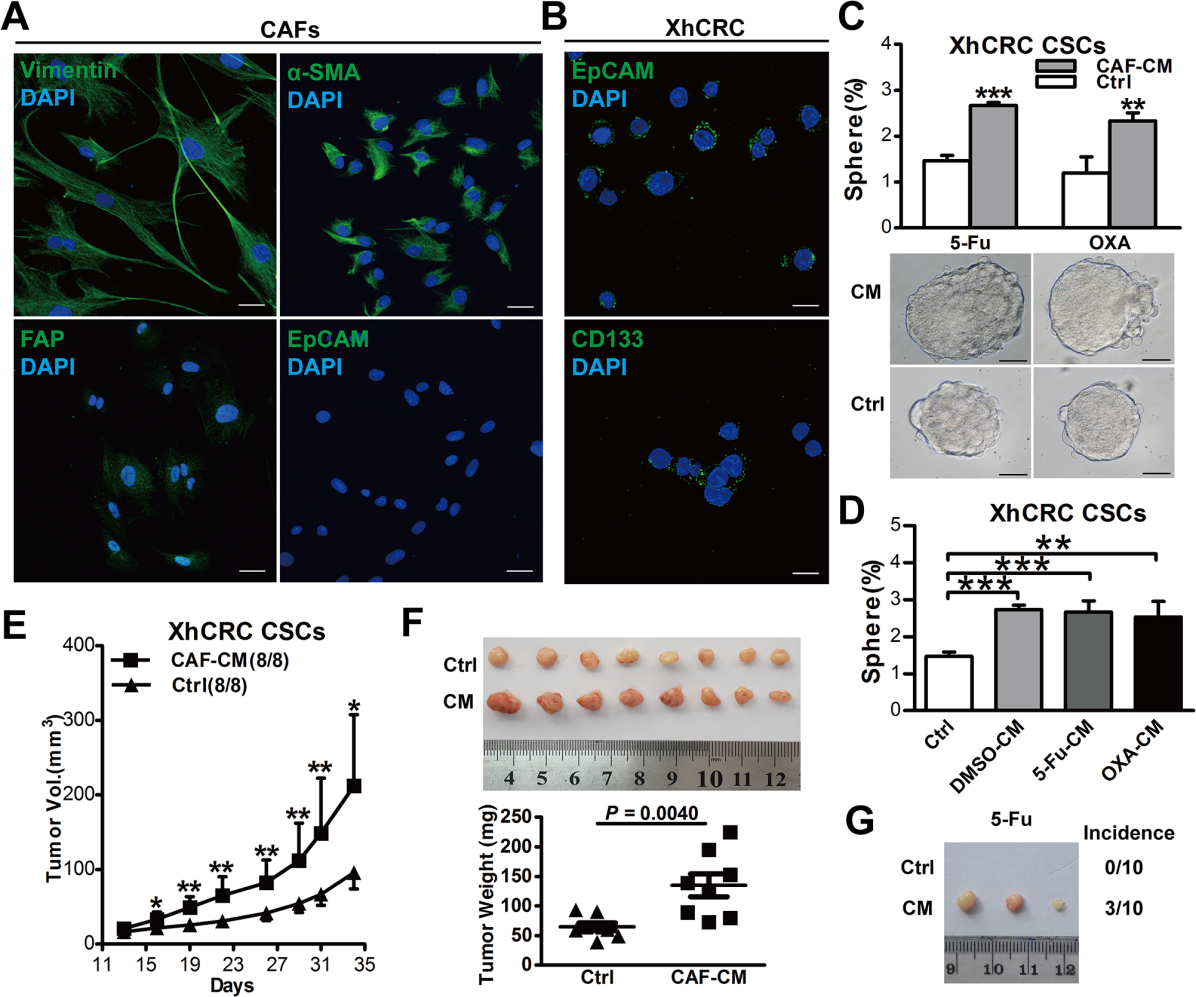
CAFs prime XhCRC CSCs to be more drug resistance through paracrine pathway. (A) CAFs derived from patient specimen were validated by positive immunostaining for CAF markers (α-SMA,Vimentin and FAP) and negative immunostaining for an epithelial marker (EpCAM). Scale bars, 30μm. (B) XhCRC CSCs were validated by positive immunostaining for epithelial marker (EpCAM) and CSC marker (CD133), Scale bars, 10μm. (C) Sphere-forming capacity of XhCRC CSCs in CAFs-derived conditioned media (e.g., 5-FU, OXA, DMSO-treated CAFs). (D) Sphere-forming capacity of XhCRC CSCs treated with CAF-derived CM during chemotherapy (5-Fu or OXA). Representative microscopic images are shown. Scale bars, 50μm. (E, F) CAF-derived CM affected on tumor growth of XhCRC CSCs in female NOD/SCID mice treated with OXA. Tumor growth curves are shown in E, Shown in F are tumor weights and images at the end of experiments. Data are presented as mean ± SD; *P< 0.1; **P< 0.05. (G)CAF-derived CM affected on tumor growth of XhCRC CSCs transplanted in female NOD/SCID mice upon treatment with 5-Fu.

To explore whether CAFs prime CSCs isolated from patient-derived xenograft (XhCRC) thus contributing to drug resistance. We first separated and cultured primary carcinoma-associated fibroblasts (CAFs) from a female patient with Duke B colorectal adenocarcinoma, and immunostaining confirmed that these cells were positive for fibroblast markers such as vimentin [22], α-SMA [23] and FAP [24] and negative for epithelial cell marker EpCAM [25] (Fig 4A). We then harvested conditioned medium from cultured CAFs (CAF-CM) and performed sphere-formation assays with CAF-CM or control medium in XhCRC CSCs. Consistent with findings in SW620 CSCs, CAF-CM also promoted sphere-generating capacity of XhCRC CSCs and protected them from chemotherapy (5-Fu or OXA) (Fig 4C, *P*<0.001 in 5-Fu group, *P*<0.01 in OXA group). As previously reported, chemotherapy could not only induce cancer cell apoptosis but also alter tumor microenvironment in solid tumors [20, 26]. To evaluate whether chemotherapy makes some differences in tumor-inducing effects of CAFs, we treated CAFs with 5-Fu/OXA or DMSO for 12 hours, and after rinsing chemotherapeutic agents, conditioned medium was harvested as described in *Materials and Methods*. However, our data revealed that there was no significant difference between chemotherapy-treated CAFs and DMSO-treated CAFs, all CAF-CMs could enhance sphere-forming capacity of XhCRC CSCs (Fig 4D, *P*<0.001 DMSO-CM/5-Fu vs. control, *P*<0.01 OXA-CM vs. control), implying that CAFs already prime CSCs through paracrine manner prior to chemotherapy. To further explore the effects of CAF-CM on tumor growth of XhCRC CSCs, we subcutaneously injected 50,000 XhCRC CSCs into bilateral backs of female NOD/SCID mice (n = 4), which simultaneously received OXA and CAF-CM. Consistent with *in vitro* findings, CAF-CM-treated XhCRC CSCs generated faster-growing and larger tumors when compared with control medium (Fig 4E and 4F). Similarly, upon treatment with 5-Fu, CAF-CM-treated XhCRC CSCs initiated more tumors when compared with control medium (Fig 4G).

Collectively, these results clearly demonstrate that CAFs prime CSCs and thus promote chemoresistance in colorectal cancer via secreting soluble factor(s).

### Fibroblasts-derived exosomes prime CSCs to be more chemoresistance

**Fig 5:**
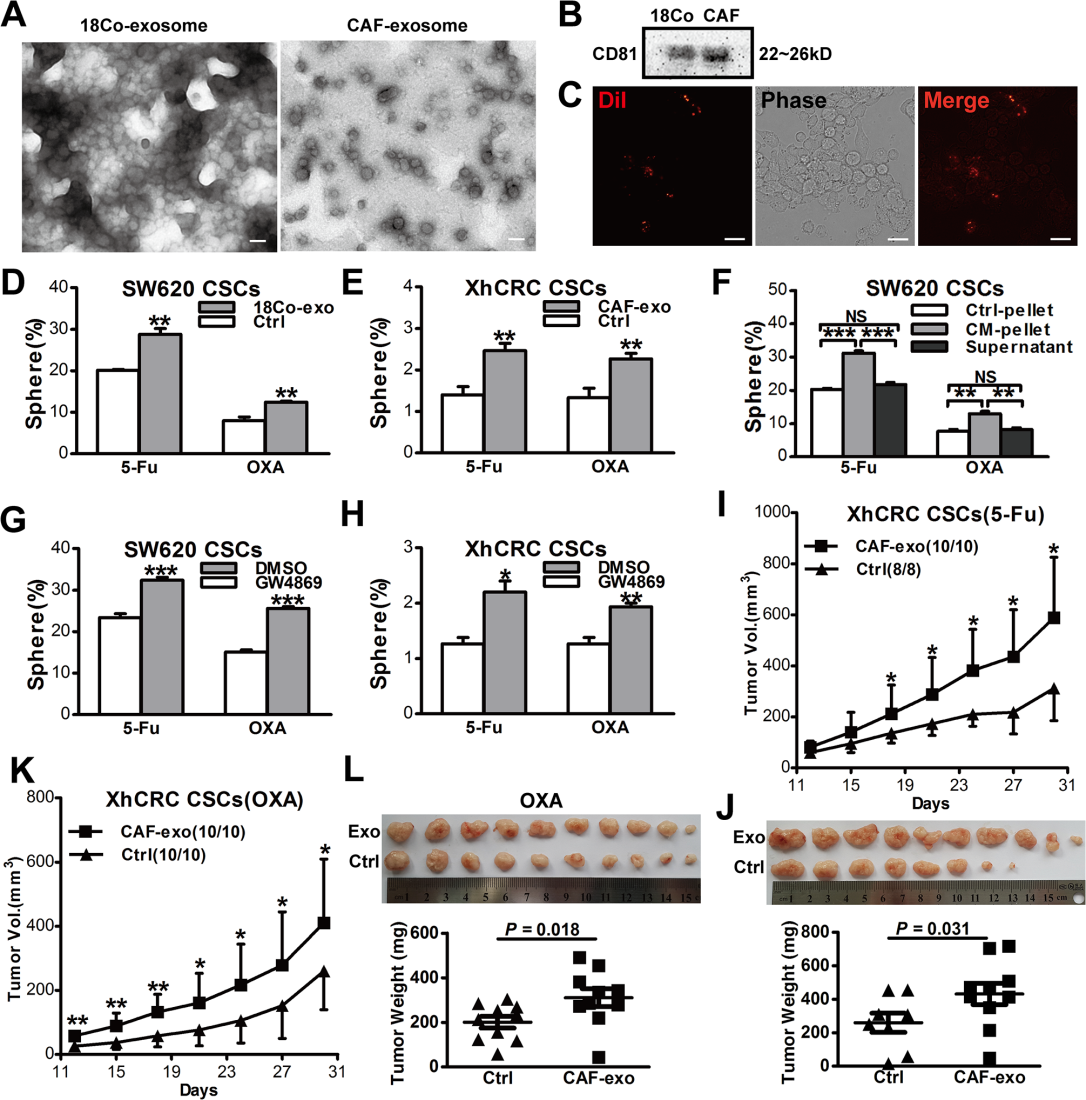
Fibroblast-derived exosomes prime CSCs to be more chemoresistance. (A) Transmission electron microscopic image of the exosomes derived from 18Co cells and CAFs. Scale bars, 100nm. (B) Western blotting of CD81 in exosomes. (C) Representative microscopy of SW620 cells exposed to DiI-labeled exosomes for 12h. (D, E) Sphere-forming capacity of SW620 or XhCRC CSCs treated with 18Co/CAF-derived exosomes during chemotherapy (5-Fu or OXA). (F) Sphere-forming capacity of SW620 CSCs treated with ultracentrifugation-purified 18Co-derived exosomes during chemotherapy (5-Fu or OXA). (G, H)Fibroblasts were treated with 10mM GW4869 (dissolves in DMSO) for 24h. The CMs derived from GW4869/DMSO-pretreated fibroblasts were added to CSCs. (I-L) CAF-derived exosomes affected on tumor growth of XhCRC CSCs in female NOD/SCID mice treated with 5-Fu or OXA. Tumor growth curves are shown in I(5-Fu) and K(OXA), and tumor weights and images at the end of experiments are shown in L(5-Fu) and J(OXA). Data are presented as mean ± SD; *P< 0.1; **P< 0.05.

Recent evidence suggests that exosomes, soluble factors which are secreted by a variety of cells, have been implicated in metastasis and drug resistance [8, 27, 28]. We hypothesized that exosomes might also contribute to drug resistance in our experimental system. Therefore, we first purified exosomes from 18Co-CM and CAF-CM as described in *Materials and Methods*, and then confirmed their structural nature under phase-contrast electron microscopy (Fig 5A) and by immunoblotting of exosome marker protein CD81 (Fig 5B). To investigate whether fibroblast-secreted exosomes can transfer to CRC cells, we labeled exosomes with Dil, a lipophilic fluorescentcarbocyanine dye. We observed that Dil-labeled exosomes derived from 18Co cells were taken up by SW620 cells after 12 hours co-incubation (Fig 5C). We then treated CSCs with purified exosomes instead of CM, and found that both SW620 and XhCRC CSCs treated with exosomes generated more spheres (*P*<0.01) (Fig 5D and 5E), suggesting that exosomes, which are derived from fibroblasts, may prime CSCs to become more chemoresistance. To further confirm that fibroblast-secreted exosomes rather than other soluble factors take the above effects, we adopted standard differential ultracentrifugation, instead of Total Exosome Isolation Kit, to isolate exosomes. Similar to kit-purified exosomes, CM-pellet-treated SW620 CSCs formed more spheres when compared with control pellets (*P*<0.001 in 5-Fu group, *P*<0.01 in OXA group), and the exosome-depleted supernatant from 18Co-CM did not take this effect (*NS control pellet vs supernatant*) (Fig 5F). To further confirm whether fibroblast-derived exosomes mediate in drug resistance, we treated fibroblasts (18Co and CAFs) with GW4869, a specific neutral sphingomyelinase (nSMase) inhibitor which blocking exosomes release, and then obtained the CM (i.e., exosome-depleted CM). Consistent with the previous findings, exosome-depleted CM remarkably decreased chemoresistance of CSCs (Fig 5G, *P*<0.001, 5H, in 5-Fu group, *P*<0.001 in OXA group). In addition, *in vivo* experiments also showed that XhCRC CSCs, while treated with CAF-derived exosomes, generated faster-growing (Fig 5I and 5K) and larger tumors (*P*<0.05) (Fig 5J and 5L) during chemotherapy (5-Fu or OXA). These data clearly showed that fibroblasts-derived exosomes primed CSCs to be more drug resistance.

### Exosomes prime CSCs through Wnt signaling pathway

It has been previously shown that Wnt signaling activity is a functional marker for cancer stem cells and is crucially important in maintaining stemness in colon cancers. In a similar manner to normal intestinal stem cells, Wnt activity is not merely a cell-intrinsic feature that can be used to define the colon cancer stem cell (CSC) population, but it is also regulated by the extrinsic microenvironment [29–32]. Wnt activity is associates with the T-cell factor/lymphoid enhancer factor (TCF/LEF) family of transcription factors which activating specific Wnt target genes [33, 34].

**Fig 6:**
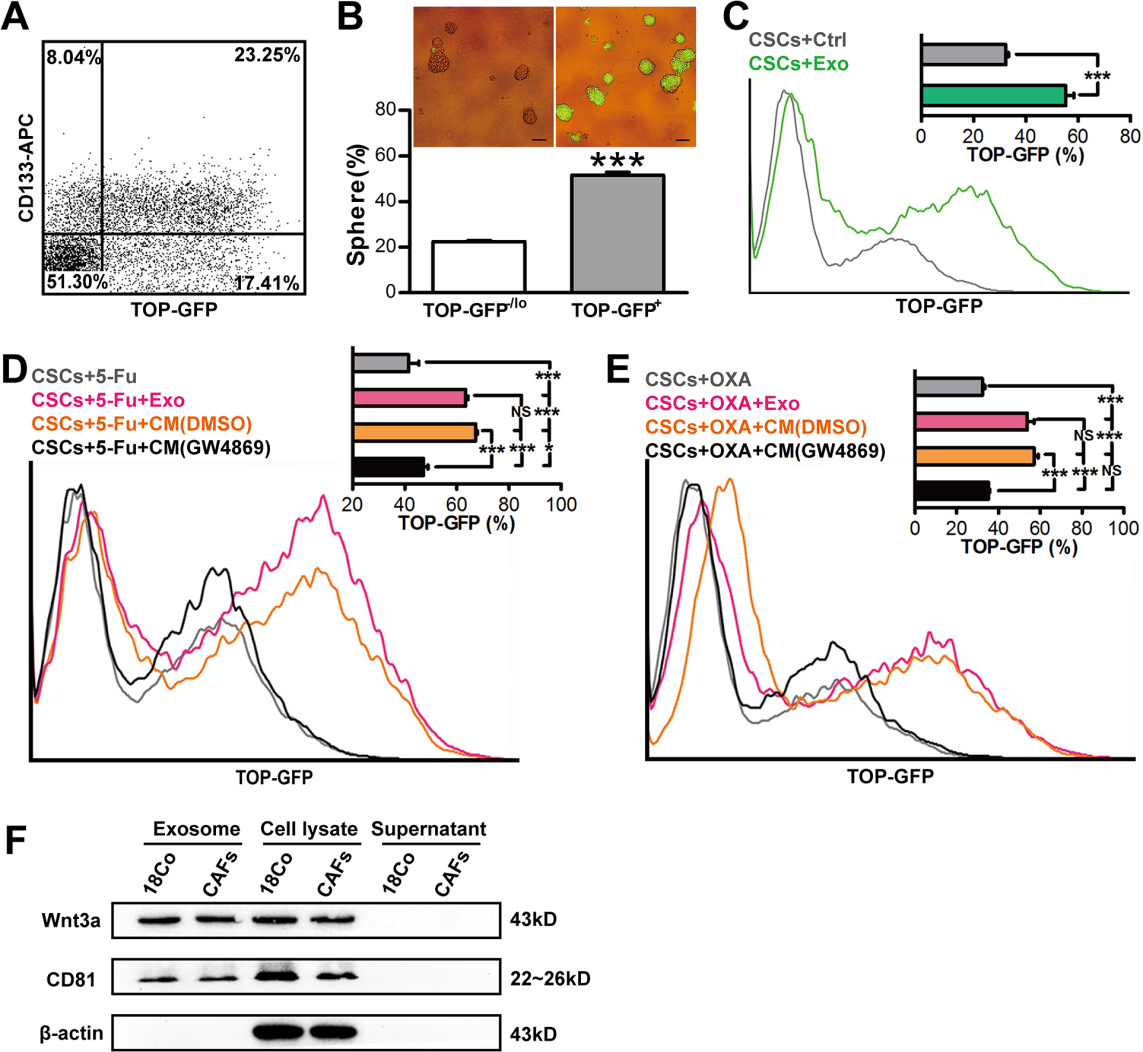
Exosomes prime CSCs through mediating Wnt activity. (A) TOP–GFP levels are associated with CSC maker expression. (B) Sphere-forming efficiency and representative spheres (insert) of TOP-GFP^+^ and TOP-GFP^-/lo^ SW620 cells. ^***^ *P*<0.001. (C)Representative flow cytometry and quantification (insert) of TOP-GFP expression of CSCs treated with 18Co-derived exosomes (green line).Gray line represents the control group.(D, E) Representative flow cytometry and quantification (insert) of TOP-GFP expression of CSCs treated with 18Co-derived exosomes (magenta line) or GW4869 (black line)/DMSO (orange line)-pretreated 18Co-CM upon chemotherapy (5-Fu or OXA). Gray line represents the control group. (F) Fibroblast-derived exosomes were purified by ultracentrifugation. The pellet, supernatant and fibroblast cell lysate were subjected to western blotting for exosome maker CD81 and Wnt3a.

To interrogate whether exosomes prime CSCs via Wnt signaling pathway, we infected SW620 cells with the lentivirus, in which TCF/LEF reporter drives expression of GFP(TOP-GFP) and found that a strong correlation existed between expression of GFP and CD133: ~74% CD133^+^ cells are GFP^+^ (i.e., Wnt-high) and ~57% GFP^+^ cells are positive for CD133 (Fig 6A).To further investigate whether GFP^+^ cells highly possess self-renewing capacity, we purified GFP^+^ (i.e., WNT^+^) and GFP^-/lo^ cells (i.e., WNT^-/lo^) and carried out sphere formation assay. Consistent with the previous study [21], GFP^+^ SW620 cells generated more spheres than GFP^-/lo^ cells (*P*<0.001) (Fig 6B), suggesting that GFP expression levels are positively correlated with self-renewing capacity. To further confirm that exosomes prime CSCs through Wnt signaling, we infected CD133^+^ SW620 cells (i.e., CSCs) with TOP-GFP lentivirus, and then treated with 18Co-exosomes for three days. As demonstrated in Fig 6C, 18Co-exosomes significantly enhanced Wnt activity (*P*<0.001). We then treated SW620 CSCs, which simultaneously infected with TOP-GFP lentivirus, with 18Co-exosomes or GW4869/DMSO-pretreated 18Co-CM upon administration of 5-Fu or OXA. Three days later, we evaluated GFP expression by flow cytometry and found that both 18Co-exosomes and 18Co-CM significantly increased GFP expression during chemotherapy (*P*<0.001), whereas depletion of exosomes by GW4869 remarkably decreased GFP expression (*P*<0.001) (Fig 6D and 6E). Moreover, we found that Wnt3a, which is a critical ligand to activate Wnt pathways via paracrine signaling, was detectable in both fibroblasts and fibroblast-derived exosomes, and more significantly, Wnt3a was undetectable in the supernatant (Fig 6F). These data clearly showed that fibroblast-derived exosomes primed CSCs to become more drug resistance via Wnt signaling pathway.

## Discussion

In this study, our data suggests that two underlying mechanisms may cooperate and lead to drug resistance of CSCs in the recurrent CRC tumors. One is that CSCs are inherently resistant to chemotherapy; another one is that stromal fibroblasts secrete exosomes, which in turn prime CSCs hereby leading to be more drug resistance.

First, we have shown that in our experimental system, consistent with the findings in previous studies [13, 35], CD133^+^ cells may enrich putative CSCs manifesting as enhanced sphere-forming capacity when compared with CD133^-/lo^ cells in SW620 and xenograft cells (Fig 1). Nevertheless, one recent study has shown that none of CSC markers, such as CD44, CD133 and aldehyde dehydrogenase (ALDH), consistently enrich CSCs in CRC established cell lines such as HCT116, HT29 and SW480 [36]. However, it is hard to read out the difference between distinct cell populations when high cell dosage of established cell lines (i.e., 1,000 and 10,000/injection) was applied in tumorigenic assays. Indeed, none of CSC marker can universally read out CSCs [37], and CSCs must be functionally defined by sphere formation assay and *in vivo* transplantations. In our experimental system, purified CD133+ CRC cells were able to be passaged under sphere-forming assay for at least three generations, and more significantly, CD133^+^ CRC cells highly enriched for tumorigenic cells in transplantation assay, suggesting that CD133^+^ CRC cells may enrich for putative CSCs. It has been demonstrated that CSCs are inherently resistant to cell death upon chemotherapy [13]. Indeed, our findings also revealed that percentage of CD133^+^ cells in bulk CRC cells significantly increased after chemotherapy (i.e., 5-Fu and OXA) (Fig 2C and 2D), and furthermore, purified CD133^+^ cells are more resistant to chemotherapy-induced cell death (Fig 2A).

Second, we have shown that conditioned medium, which are derived from both fibroblast cell line (18Co) and CAFs without treatment with chemotherapeutic agents, promote drug resistance of CSCs in CRC cell lines and xenografts, both *in vitro* and *in vivo* (Figs 3 and 4). Furthermore, our data has also shown that any of the conditioned medium which derived from DMSO-, 5-Fu- or OXA-treated CAFs, is able to promote sphere-forming capacity of CSCs (Fig 4), coupled with the findings of the recent study in which both naïve and chemotherapy-treated CAFs promote CSC growth via paracrine signaling [20], suggesting that CAFs, while treated or untreated with chemotherapeutic agents, may both contribute to drug resistance via ‘soluble’ factors (soluble factor-mediated drug resistance, SFM-DR). Moreover, another experimental system (i.e., TOP-GFP), which based on the activity of Wnt signaling [21], was also applied in our studies. Using this experimental system, we also demonstrated that CAFs prime CSCs through paracrine fashion thus contributing to drug resistance (Fig 6).

Finally, our studies implicate that fibroblasts secret exosomes and thus promote drug resistance. In support, exosomes can be purified from the CM derived from both 18Co cells and primary CAFs (Fig 5A). In addition, purified exosomes promote sphere-formation and tumorigenic capability of the CSCs (Fig 5D–5F and 5I and 5J). Finally, inhibition of exosome release blocks the above effect (Fig 5G and 5H), and more significantly, blocking exosome release also decreases the number of CSCs (Fig 6C–6E). Exosomes are membrane-enclosed vesicles secreted by cells and play complex roles in intercellular communication. Exosomes may act as natural vehicles for delivering protein, mRNA, or microRNA to recipient cells [38–41] to mediate their biological and pathological functions such as metastasis-promoting effects [42]. A variety of cells may secret exosomes. For example, melanoma cells secret exosomes to ‘educate’ bone marrow progenitor cells and promote metastasis [43]. In the present study, we show that exosomes, derived from fibroblasts, can promote drug resistance via priming CSCs, even before administration of chemotherapeutic agents.

Altogether, our results provide rationale that novel therapeutics targeting CAFs before administration of chemotherapy should be developed to gain maximal clinical benefits.
